# Supplementary material for: Machine-learning and mechanistic modeling of metastatic breast cancer after neoadjuvant treatment
Source: PLoS Comput Biol. 2024 May 3;20(5):e1012088. doi: 10.1371/journal.pcbi.1012088 (PMC11095706; doi:10.1371/journal.pcbi.1012088)
Supplement: S1 Table — Animal groups showing treatment schedules and dosing during a presurgical neoadjuvant period of 14 days. (PDF) [file pcbi.1012088.s001.pdf]

Table S1:

Group	N	Duration (Days) Sunitinib (mg/kg/day)			Surgery (DPI)	Modeling	Abbrev.
		120	60	0			
**1**	6	0	0	14	38	Training	Veh.
**2**	21	0	0	14	34	Training	Vech.
**3**	21	0	14	0	34	Training	Su60(14D)
**4**	6	0	7	7	34	Training	Su60(7D)
**5**	15	0	3	11	34	Training	Su60(3D)
**6**	15	3	11	0	34	Training	Su120(3D)/Su60(11D)
**7**	20	3	0	11	34	Training	Su120(3D)
**8**	6	3	11	0	38	Validation	Su120(3D)/Su60(11D)
**9**	6	3	8	3	38	Validation	Su120(3D)/Su60(8D)
**10**	6	3	4	7	38	Validation	Su120(3D)/Su60(4D)
**11**	6	3	0	11	38	Validation	Su120(3D)

Table S1: Animal groups showing treatment schedules and dosing during a presurgical neoadjuvant period of 14 days.
