# Supplementary material for: Machine-learning and mechanistic modeling of metastatic breast cancer after neoadjuvant treatment
Source: PLoS Comput Biol. 2024 May 3;20(5):e1012088. doi: 10.1371/journal.pcbi.1012088 (PMC11095706; doi:10.1371/journal.pcbi.1012088)

Figure S1. Comparison of simulation of therapy (A) vs no therapy (B) on metastases

Surgery at day 34

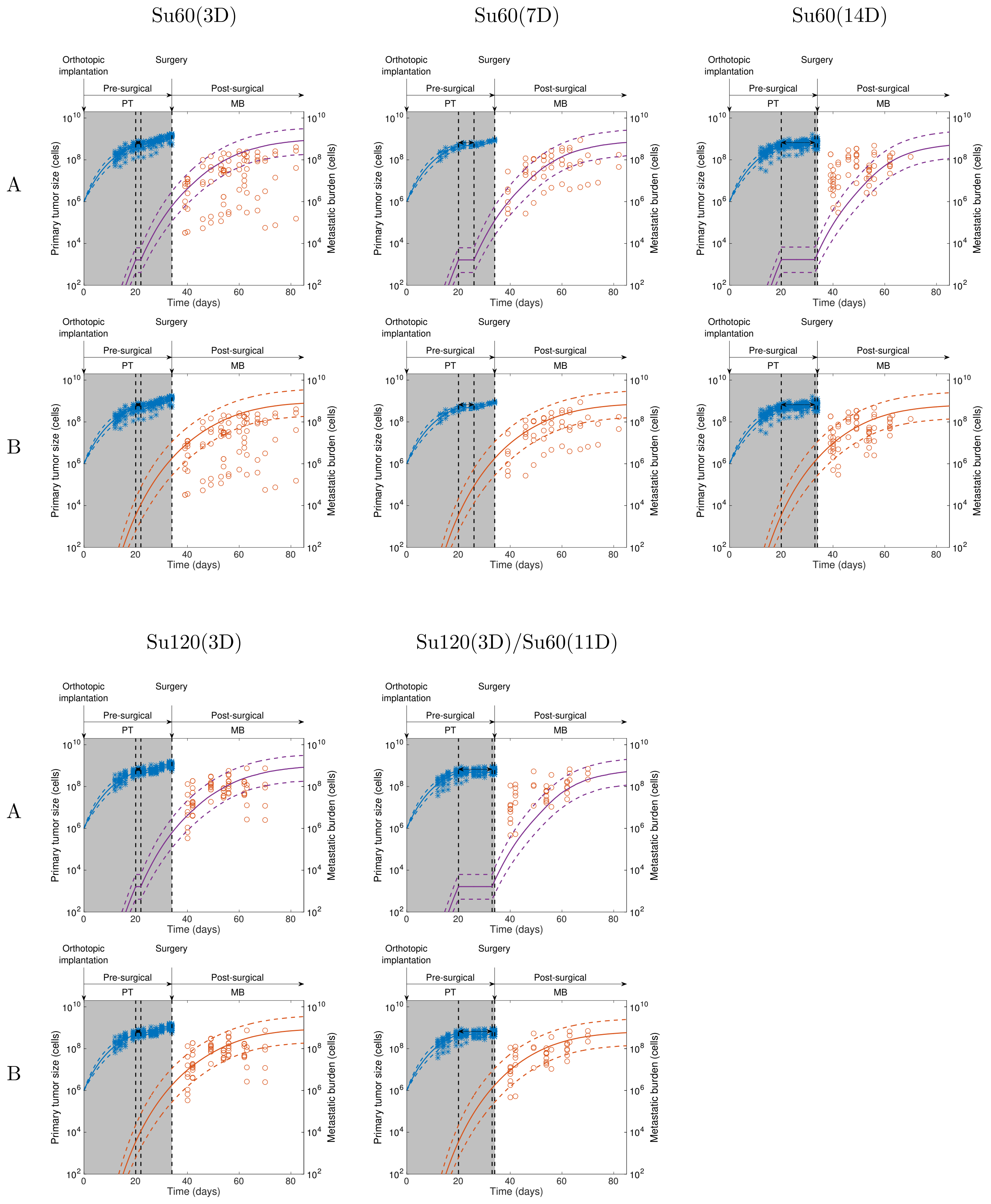

Surgery at day 38

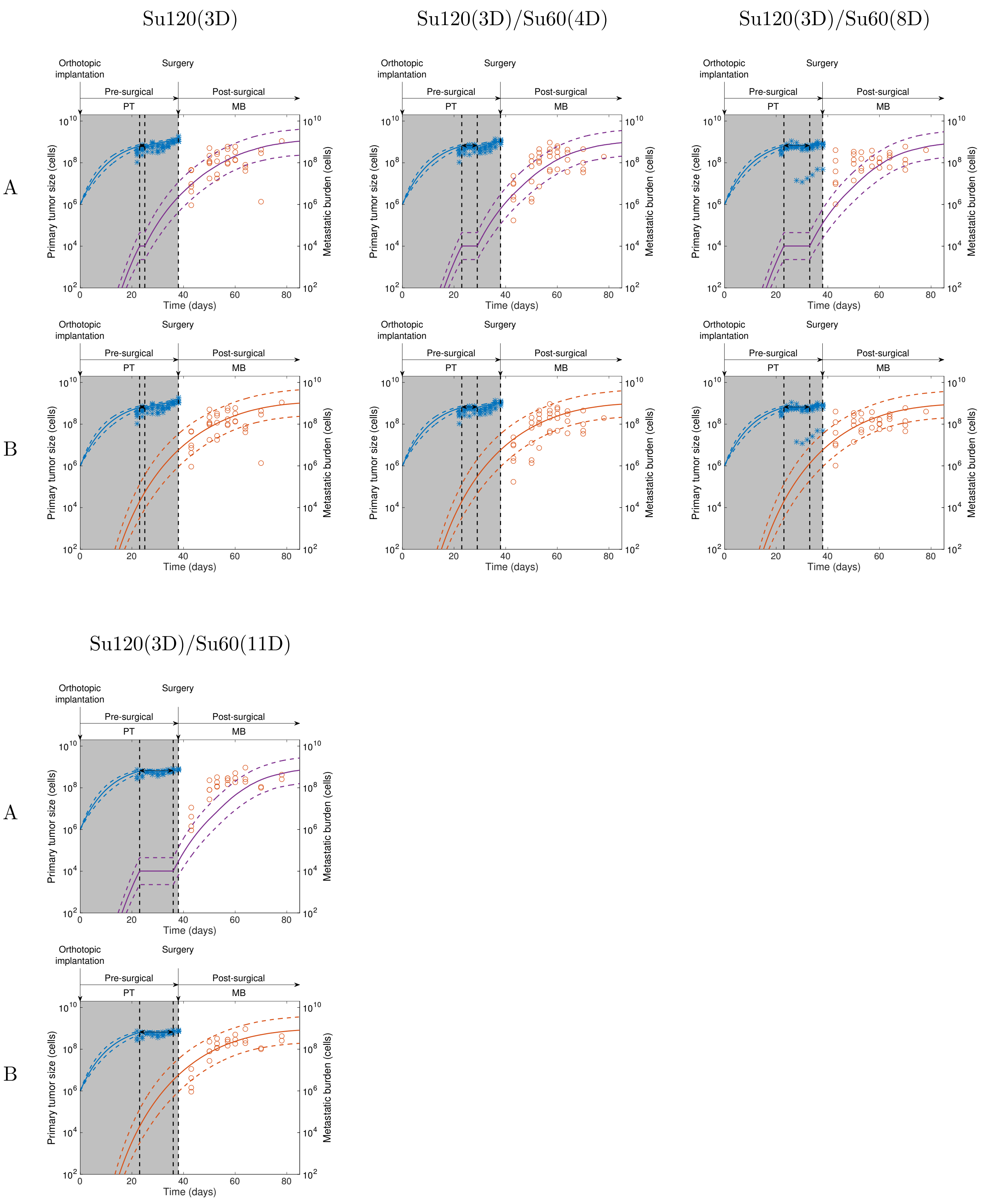

Supplement: S1 Fig — (PDF) [file pcbi.1012088.s002.pdf]
