# Supplementary material for: Machine-learning and mechanistic modeling of metastatic breast cancer after neoadjuvant treatment
Source: PLoS Comput Biol. 2024 May 3;20(5):e1012088. doi: 10.1371/journal.pcbi.1012088 (PMC11095706; doi:10.1371/journal.pcbi.1012088)

Figure S2. Population fits of all the groups used to calibrate the K-PD model parameters (surgery at day 34)

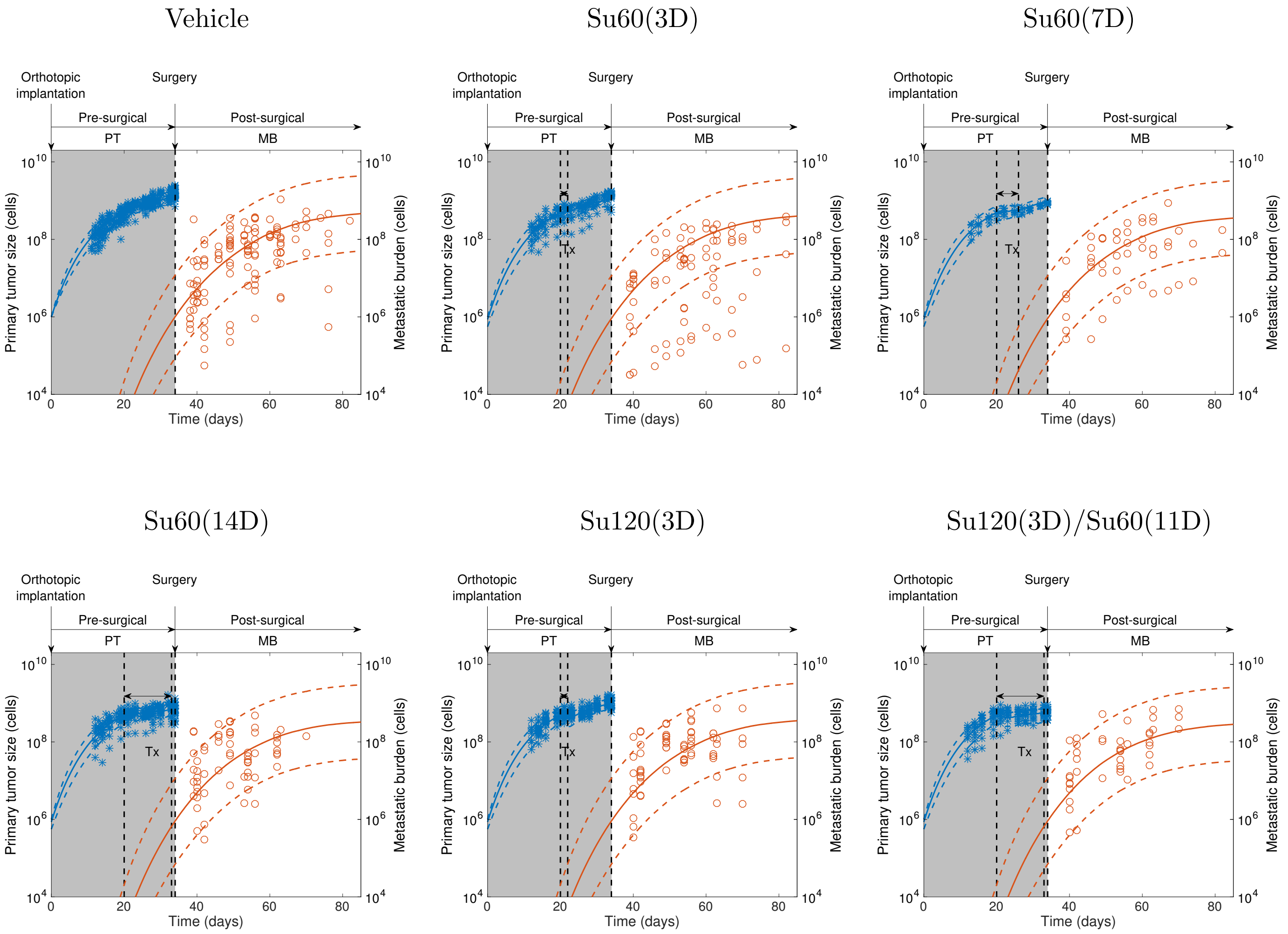

Supplement: S2 Fig — (PDF) [file pcbi.1012088.s003.pdf]
