# Supplementary figures and images for: Machine-learning and mechanistic modeling of metastatic breast cancer after neoadjuvant treatment
Source: PLoS Comput Biol. 2024 May 3;20(5):e1012088. doi: 10.1371/journal.pcbi.1012088 (PMC11095706; doi:10.1371/journal.pcbi.1012088)

**Figure S3. Representative individual fits of the K-PD model for Sunitinib-treated animals**

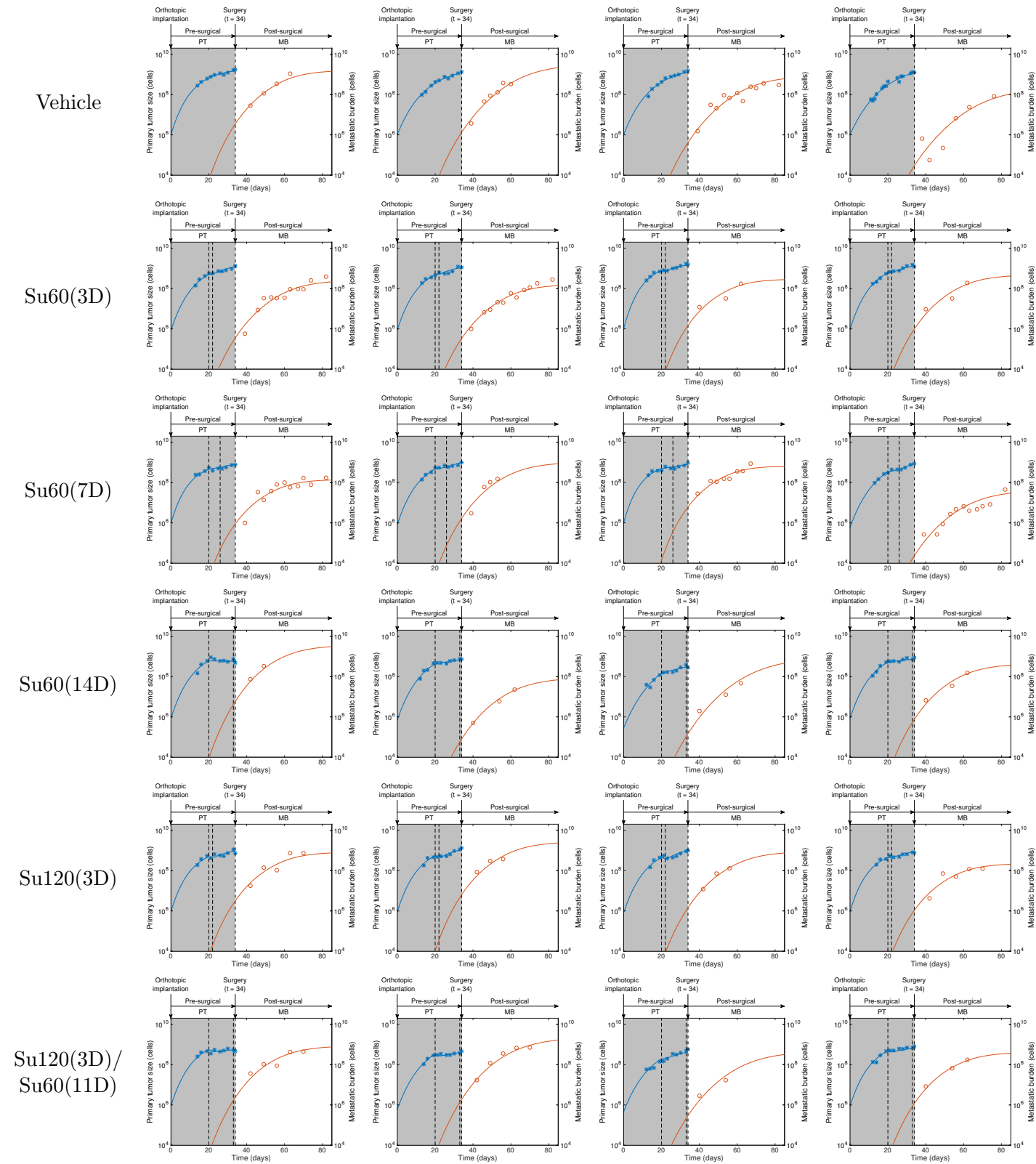

Supplement: S3 Fig — (PDF) [file pcbi.1012088.s004.pdf]

Figure S4. K-PD model predictions in independent datasets (surgery at day 38)

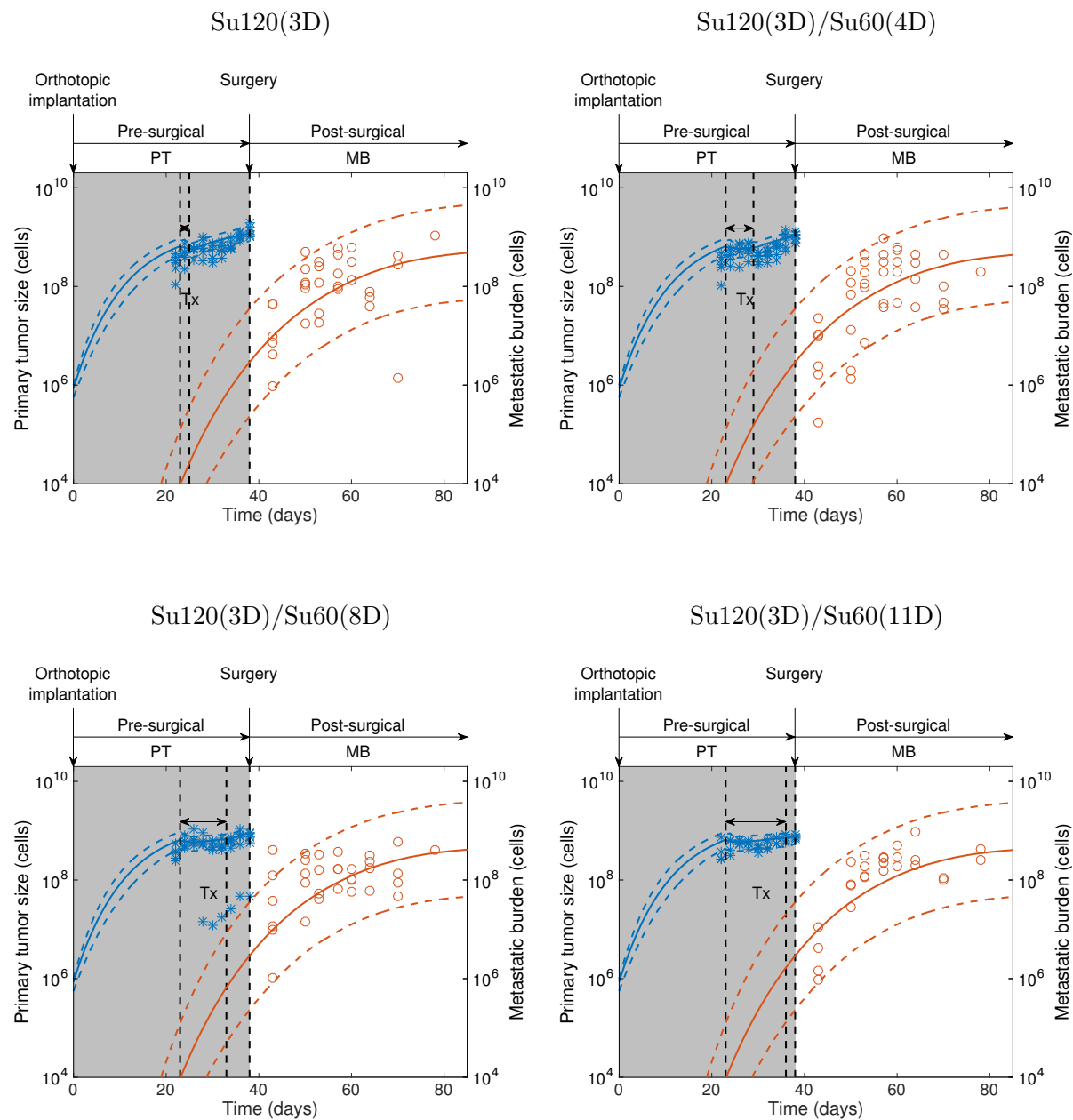

Supplement: S4 Fig — (PDF) [file pcbi.1012088.s005.pdf]

**Figure S6. Distribution of the individual parameters**

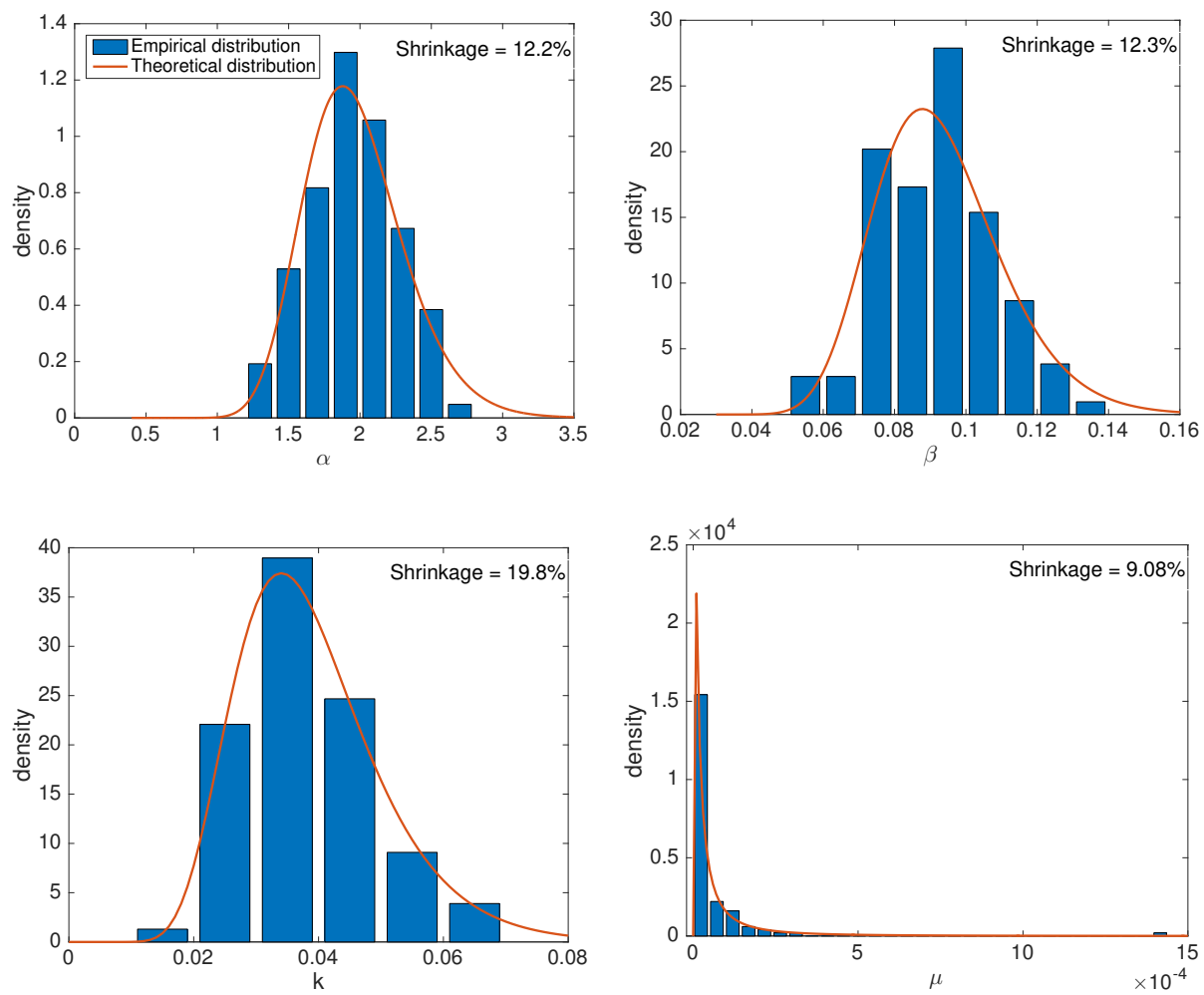

Supplement: S6 Fig — (PDF) [file pcbi.1012088.s007.pdf]

Figure S7. Correlations between random effects

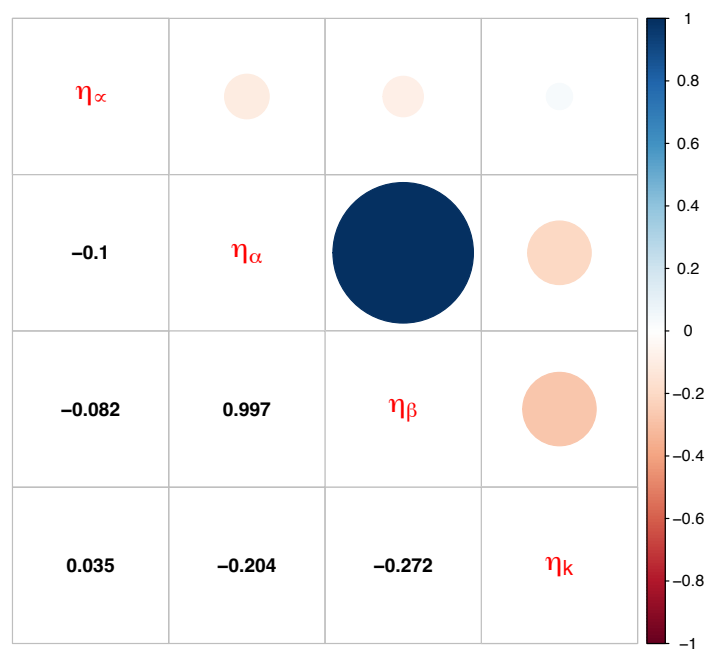

Supplement: S7 Fig — (PDF) [file pcbi.1012088.s008.pdf]

**Figure S8. Individual parameters vs covariates**

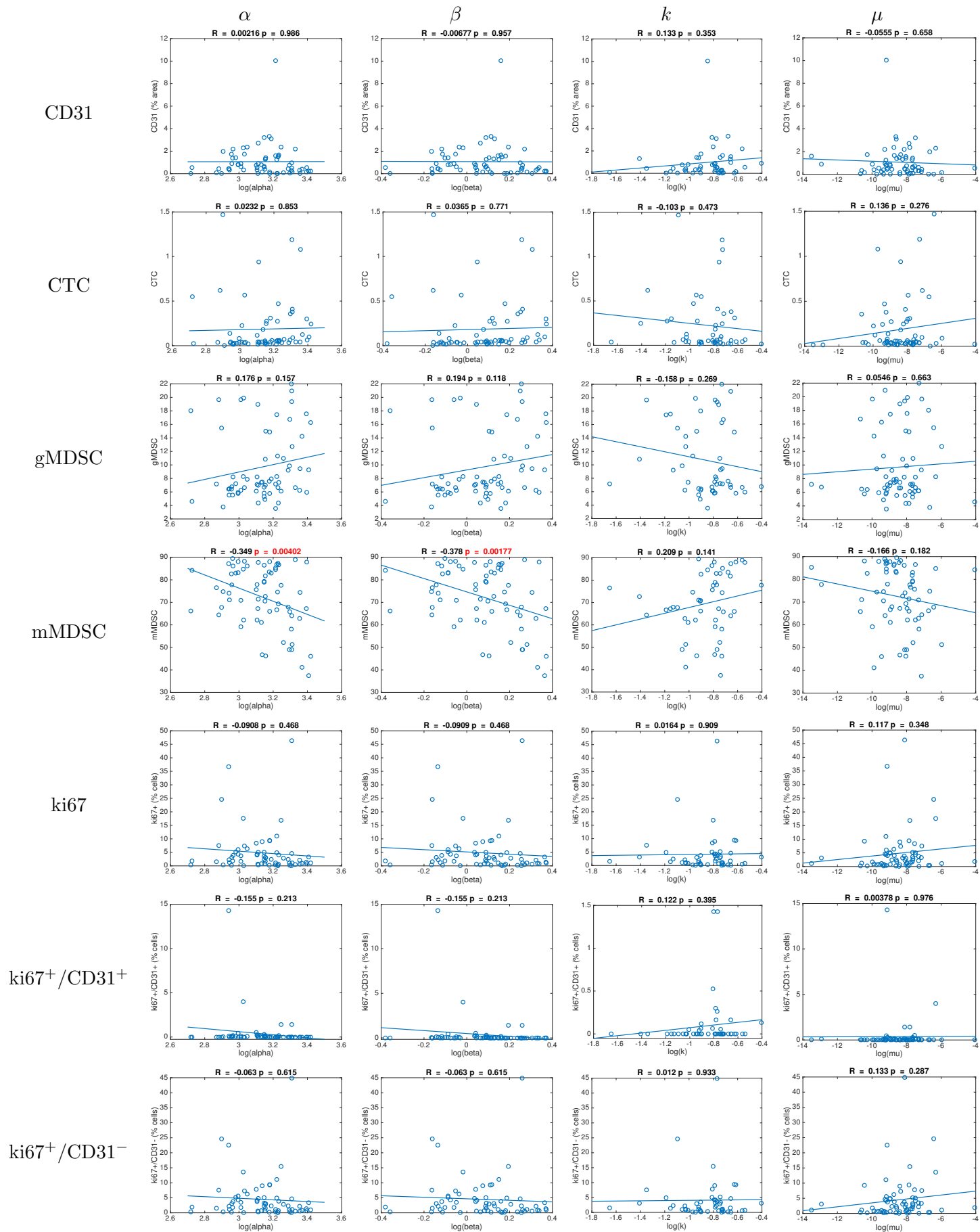

Supplement: S8 Fig — (PDF) [file pcbi.1012088.s009.pdf]
