# Supplementary material for: Machine-learning and mechanistic modeling of metastatic breast cancer after neoadjuvant treatment
Source: PLoS Comput Biol. 2024 May 3;20(5):e1012088. doi: 10.1371/journal.pcbi.1012088 (PMC11095706; doi:10.1371/journal.pcbi.1012088)
Supplement: S5 Fig — (A) Observation vs. individual prediction. Solid lines are identity lines. Dashed lines represent 90% prediction intervals. (B) Individual weighted residuals (IWRES) vs time. (C) Individual weighted residuals vs log-transformed individual predictions. (PDF) [file pcbi.1012088.s006.pdf]

Figure S5. K-PD model diagnostic plots

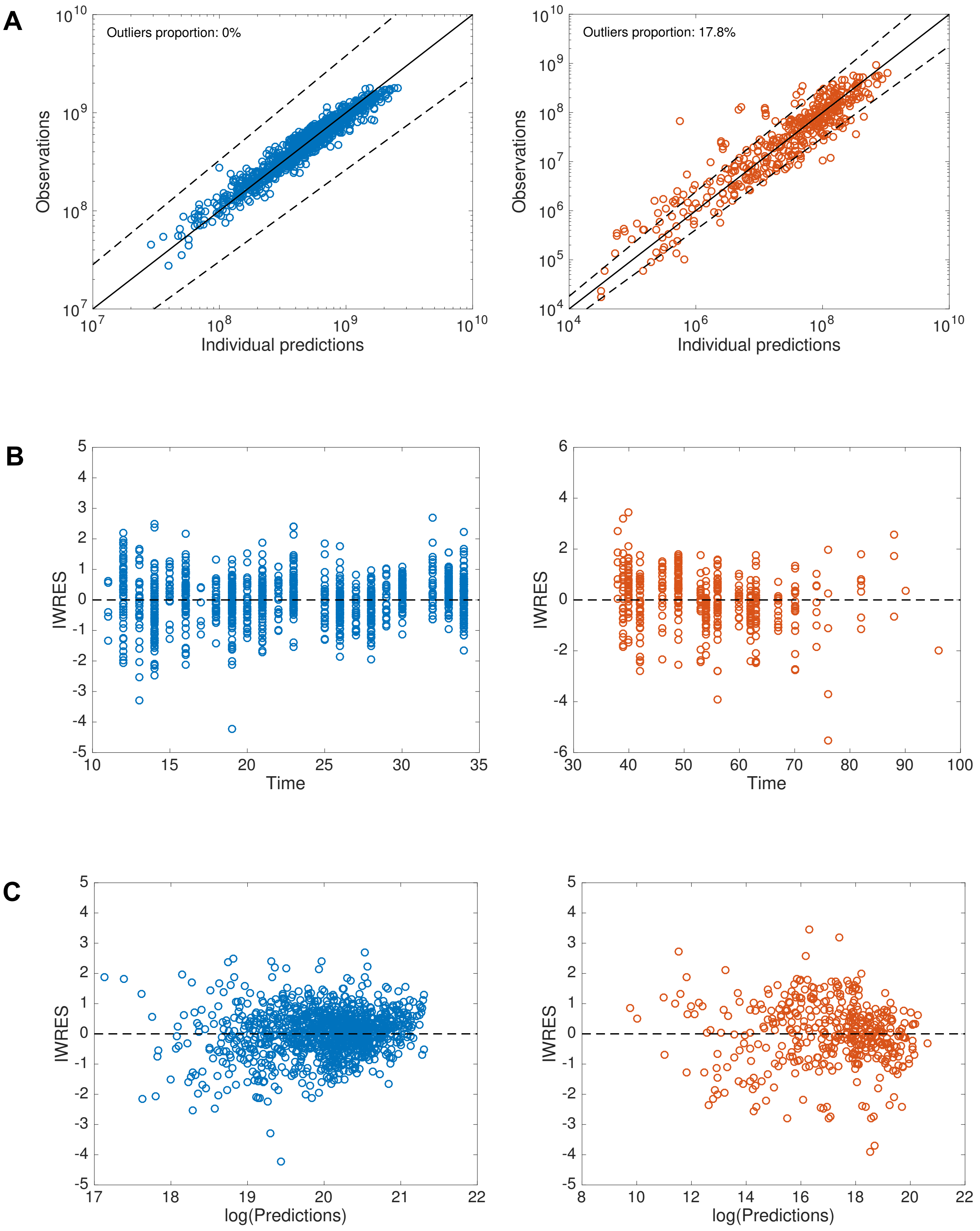

Left (blue): primary tumor, Right (red): metastatic burden.

A) Observation vs. individual prediction. Solid lines are identity lines. Dashed lines represent 90% prediction intervals.

B) Individual weighted residuals (IWRES) vs time.

C) Individual weighted residuals vs log-transformed individual predictions.
