# Supplementary material for: Machine-learning and mechanistic modeling of metastatic breast cancer after neoadjuvant treatment
Source: PLoS Comput Biol. 2024 May 3;20(5):e1012088. doi: 10.1371/journal.pcbi.1012088 (PMC11095706; doi:10.1371/journal.pcbi.1012088)

Figure S9. Observed vs Predicted values for the machine learning algorithms

Models for predicting μ

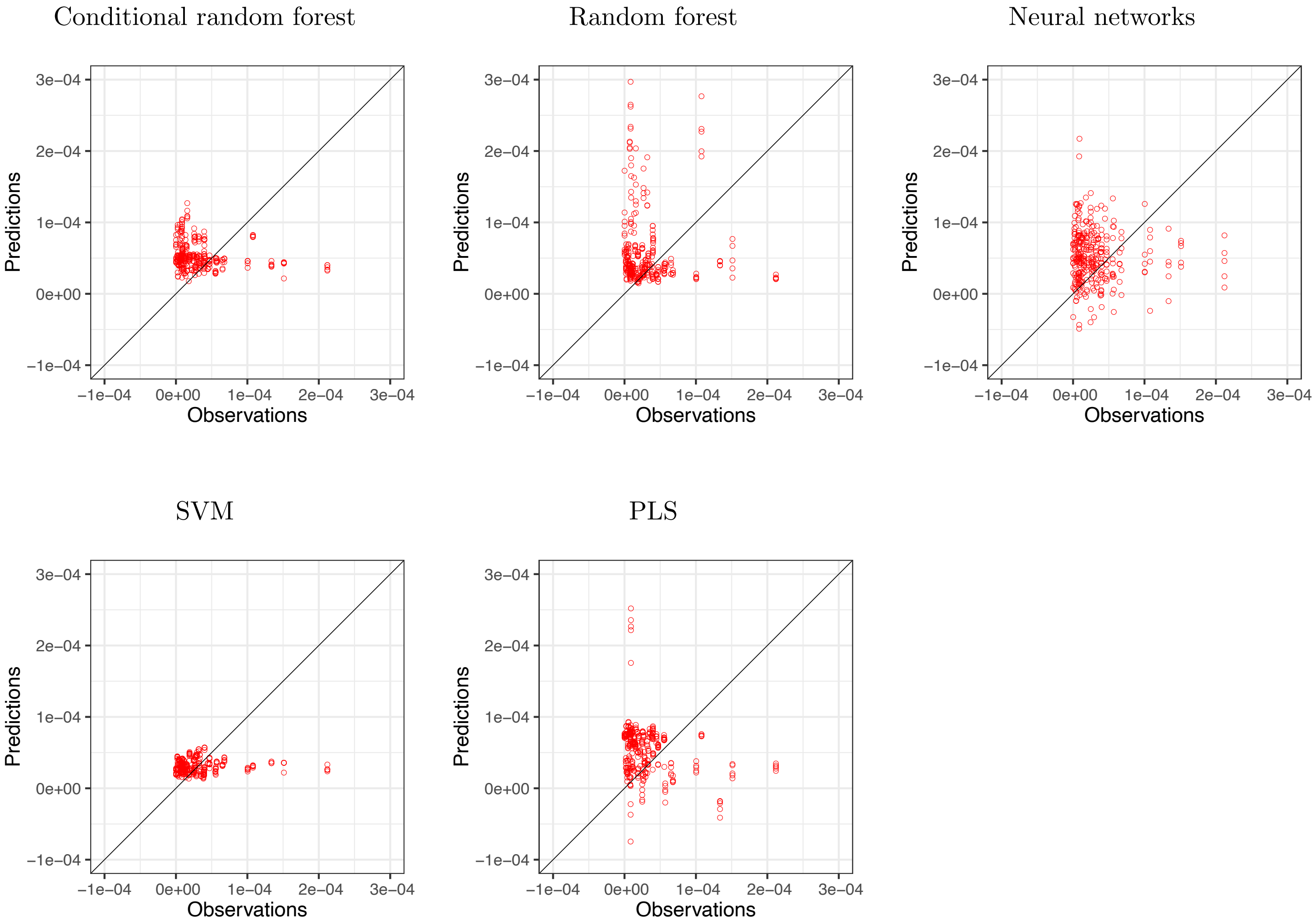

Models for predicting $\log(\mu)$

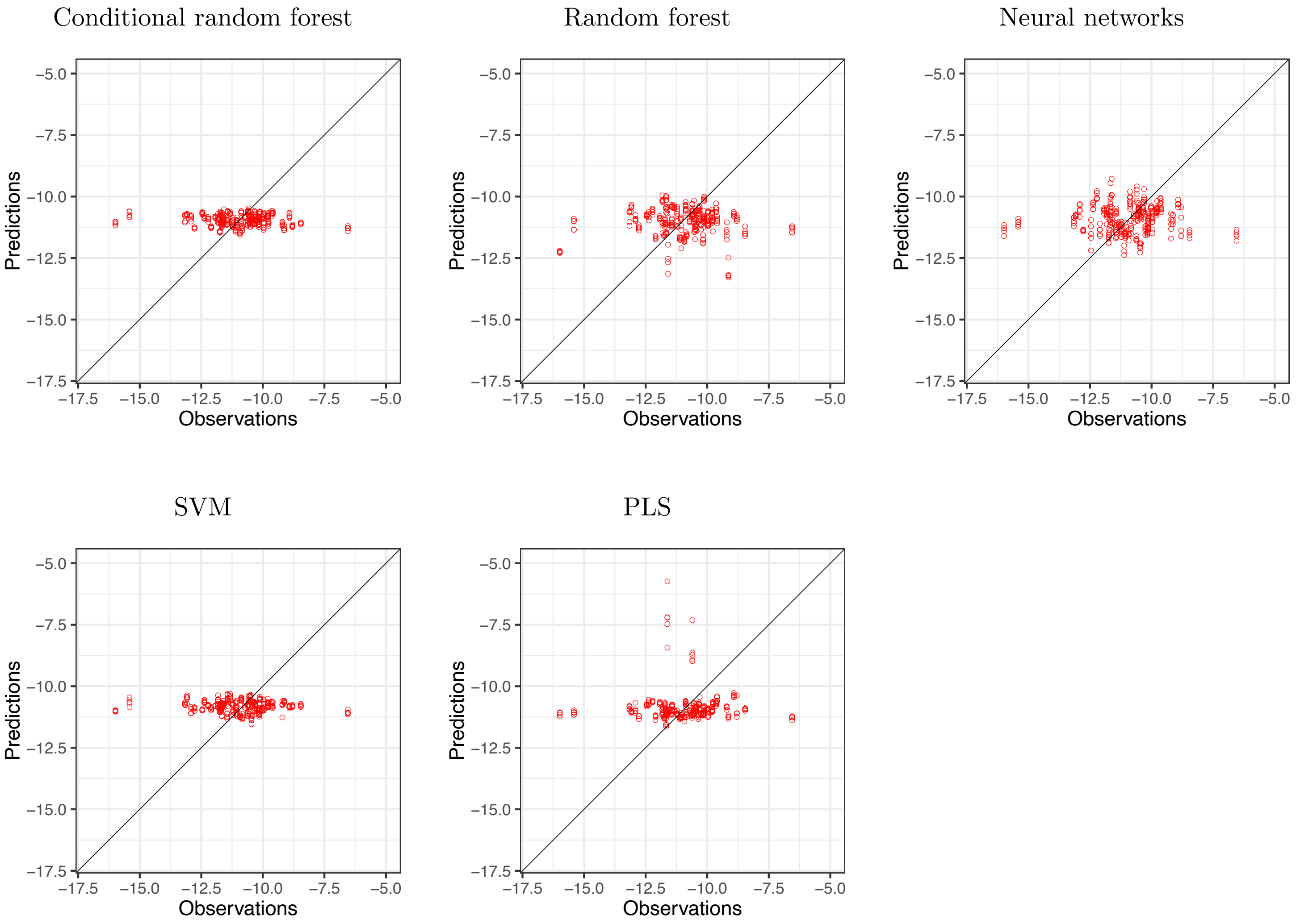

Supplement: S9 Fig — (PDF) [file pcbi.1012088.s010.pdf]
