# Supplementary material for: Machine-learning and mechanistic modeling of metastatic breast cancer after neoadjuvant treatment
Source: PLoS Comput Biol. 2024 May 3;20(5):e1012088. doi: 10.1371/journal.pcbi.1012088 (PMC11095706; doi:10.1371/journal.pcbi.1012088)
Supplement: Attachment — Submitted filename: rebuttal.docx [file pcbi.1012088.s011.docx]

Sebastien Benzekry, PhD

Head of the Inria – Inserm team COMPO

COMPutational pharmacology and clinical Oncology

Centre Inria Sophia Antipolis – Méditerranée

Centre de Recherches en Cancérologie de Marseille

Inserm U1068, CNRS UMR7258, Institut Paoli-Calmettes

Pharmacy faculty, Aix-Marseille University

**Pr P. Maini**

**The editorial office**

**PLoS Computational Biology**

Marseille, January 30, 2024

Dear Editor,

Thank you for your consideration of our work and efforts dedicated to its evaluation. We would also like to thank the reviewers for their positive appreciation of our work and valuable comments and suggestions, which helped us improve the manuscript.

Please find below a point-by-point response to the reviewers’ comments.

Sincerely,

Dr Sebastien Benzekry, Corresponding Author.

Reviewer 1

Benzekry et al present a mechanistic modeling of metastatic breast cancer after neoadjuvant treatment with sunitinib that is based on preclinical data using a mouse model of spontaneous metastasis after surgery. This work uses an extension of a previous model developed by this team in untreated animals (Ref 4). The model is sensible and the authors tested whether the neoadjuvant treatment inhibited primary tumor growth as well as post-surgery metastatic growth. In addition they used a machine-learning approach to assess the predictivity of cellular and molecular biomarkers.

The work is interesting and useful. The modeling approach is well done and sound. My main comments aim at improving and clarifying the presentation and discussion of the results.

We thank the reviewer for his/her positive appreciation and for having taken the time to evaluate our manuscript.

Major comments:

My major comments refer to the flow and the structure of the paper. Some results are given in the introduction as well as in the methods and some of the methods are in the results. More specifically:

1) The last sentence of the introduction (Lines 50-52) give results and should be removed.

Agreed and removed.

2) Lines 196-198 in the methods give the results that are also presented in the proper section (Lines 263-269 and Fig 1C). Those results should be omitted from the methods.

Agreed and removed.

3) The first section of the result should be deleted and content moved to the introduction and/or the methods as appropriate. Lines 235-242 already are in the methods (Lines 152…) or in the introduction (Lines 18-24). Lines 242-246 and Fig 1A describe the experimental protocol and should be in the methods.

Agreed and modified accordingly.

4) In the results, Lines 250-254 describe previous results and should be either in the introduction or even better in the discussion. It is actually unclear what the current results bring with respect to those previous results in term of the “differential effects with suppression of presurgical PT growth not consistently translating into reduction in postsurgical MB” as described later in this section (Lines 266-269). This should be discussed.

We have clarified this by stipulating in the introduction that the previous results had established this observation but no mechanistic explanation. The current work used mathematical modeling to simulate two biological hypotheses (Scenarii A and B) and test them against the data. This revealed that an anti-growth effect on the metastases of NATT was unlikely. This result is not straightforward as it could have been hypothesized that, the metastases being small at the time of NATT, undergoing growth arrest might not yield to a large macroscopic difference in their post-surgical development. Only a quantitative analysis such as the one we performed is able to assess the hypothesis. We have added the following sentences to the discussion to clarify:

*" Our mechanistic mathematical modeling approach offered a quantitative tool to simulate and test distinct biological scenarios, in order to distinguish the biological determinants underlying this paradoxical differential effect on the primary and distant tumors. Model predictions (with no fitting involved) allowed to disentangle the impact on MB of either PT growth arrest alone or PT and metastases growth arrest. They clearly showed: 1) that metastases growth arrest during NATT was very unlikely and 2) that the quantitative impact of PT growth suppression during NATT was sufficient to reproduce the experimental data. Furthermore, it implied a negligible reduction in dissemination, as measured by the total MB."*

5) Table 1 reports parameter estimates for a survival model. Is this the model used to predict metastatic free survival in Fig 3B? The survival model is not described in the method section. It should be.

These parameters are a residual from a previous version of the MS where we had included a survival model and are not relevant. They have been removed.

Other comments:

6) Lines 4-5: It is not the neoadjuvant trials per se that “provide several advantages to assist in novel drug development and translational research” but rather model-based analyses of those trials. The trials are designed to address unmet medical needs.

Ok. This sentence has been removed.

7) Lines 35-50: These considerations should go in the discussion. The introduction should stop at line 34.

Done.

8) Line 352: Is 4B the right figure? Or is it 4E?

Good catch, thanks. It is 4E.

9) Line 372: Fig 5 is missing. It is probably not needed, Fig S9 is fine.

Thanks for noticing. Ref to Fig 5 removed.

10) The discussion is a bit long and focuses too much on the machine learning part of the investigation… which in the end did not generate any predictive marker. The reviewer suggest to shorten this part.

Done.

11) Lines 399-403: This sentence suggests that Ref 13 is from the same team as the authors when it does not seem to be the case?

It is. Ref. 13 is first authored by John ML Ebos, last author of the current paper.

12) Lines 459-461: Are there any limitations to such investigations in preclinical models to predict clinical situations? This point may be discussed.

We discuss this in the next paragraph:

" Important limitations of our study are that we only analyzed data from one tumor type (triple negative breast cancer), one cell line in one, immune-depressed, animal system and one drug."

and have added the following sentence to emphasize limitations for translation of preclinical models to clinical situations:

" This might alter generalizability and possibly translation of preclinical findings to clinical situations."

13) Lines 475-483 seem to be describing another study that is not presented in this paper. It does not bring anything to the current discussion. The discussion being quite lengthy (see comment 10), I suggest to remove.

Agreed and removed.

Reviewer 2

The manuscript under review presents a combined experimental and computational study of the effect of neoadjuvant therapy using the tyrosine kinase inhibitor sunitinib in a surgical metastatic breast cancer mouse model. The study is very comprehensive, with 120 animals in total and a variety of different treatment/surgery schedules. First, parameters of a previously published computational model for primary and metastatic tumor growth are estimated in a mixed-effects framework. It is found that sunitinib treatment only affects the primary tumor, but not metastases. Subsequently, the authors employ various machine learning models to investigate the relationship between biomarkers determined at surgical removal of the primary tumor and individual estimates of a parameter quantifying metastatic spreading. They cannot establish any significant relationships and conclude that this preclinical model can serve as a pre-screening step for investigations of (here, unsuccessful) biomarkers in neoadjuvant trials. The study is innovative, comprehensive and has clear translational orientation towards a relevant clinical application. However, there are still some open questions and technical details to be addressed, as outlined below. Therefore, I think that the manuscript is in principle well suited for publication in PLOS Computational Biology, but some revisions are necessary first.

We thank the reviewer for his positive appreciation of our work and for the time he took to evaluate our manuscript.

Comment 1

The authors mention a survival model in the Caption of Fig 1 and in Table 1. However, it is not discussed or mentioned anywhere else. It should be part of the Methods section (including the type of hazard distribution used) and results should be discussed.

This a residual from a previous version of the MS where we had included a survival model and is not relevant anymore. It has been removed.

Comment 2

Before arriving at their metastatic model in which therapy affects the primary tumor but not metastases (model B), they investigated a model in which primary and secondary tumors are equally affected (model A). It would be instructive to investigate an intermediate model that has some effect on metastases, but not as much as the primary tumor (i.e., drug efficacy would be reduced by some factor to be estimated). In fact, although model B clearly fits the data much better than model A in Fig S1, in some panels it slightly overpredicts metastatic burden at the early timepoints (~40 days). Beyond this observation, it would be conceptually important to consider (and possibly reject) such an intermediate model, since the finding that neoadjuvant treatment always has negative consequences on metastatic relapse relies on the structure of model B.

We thank the reviewer for these observations and suggestions. However, we believe that considering intermediate models would unnecessarily complicate the narrative, while not qualitatively changing the results. That is, that there needs to be a markedly important difference between the growth suppression effects on the PT and metastases to explain the data. In addition, the second modeling step (where parameter calibration to the data was performed) partially addresses the overprediction of some data (Figure S2), by increasing the inter-animal variability of the mathematical parameters.

Comment 3

Regarding the use of machine learning to link biomarkers to predict individual model parameters, the authors currently only have a negative example with parameter mu. However, in L461-464 they also discuss a scenario with a potentially positive finding, namely the link between Ki67 and metastatic relapse. They stated that this link could be mediated through another parameter, namely alpha, which should also impact on the metastatic relapse metric that is being used. Why did the authors only try to predict mu and not alpha? Presenting a positive result would substantiate their claim of translational relevance further.

This is based on the observation that parameter mu is the main driver of inter-animal variability in metastatic development. Its coefficient of variation is estimated to 1 480%, versus 18.1% for alpha.

Comment 4

The authors briefly introduce their concept of metastatic relapse in the Results section (L319-320). After reading this part, I had no intuition about which metastatic burdens would be considered a relapse or not. Hence, the definition should be moved to the Methods section and contain additional details, in particular the cutoff value in terms of metastatic burden that results from their rationale. Potentially, this cutoff could even be integrated in some of the figures.

Our definition of metastatic relapse was unclear. We have clarified with a dedicated section in the methods.

Comment 5

In the caption of Fig. 1C, it is stated that simulations were done with a previously estimated set of model parameters based on a dataset of untreated animals. How can a treatment be simulated using such parameters? At least the drug efficacy parameter k must have been taken from somewhere else.

These simulations (and results from the first results section) *were* performed with parameters inferred from *previous data* of untreated metastatic development. As explained, the treatment effect – for this first section only – was modeled by setting the growth rates $g_{p}$ (scenario A) or $g_{p}$ and $g$ (scenario B) to zero during NATT, thus requiring no estimation of the treatment effect. This allowed precisely not to rely on any data fitting to perform the predictions which, to us, constitutes a strength of our results.

We have reorganized the methods section to clarify this point and hope this is now clearer.

Comment 6

I don’t understand Fig 3A. “Tx” seems to be constant, while “NAT duration” changes from 0 to 18 days. First, only one term should be used for treatment (say it’s NAT), consistently throughout the manuscript. Next, a varying treatment duration should result either in a varying start of treatment or varying time of surgery (it is not currently stated which of the two it is).

Figure 3A was indeed misleading. "Tx" has been changed to "NATT", also modified in Figure 3B for consistency with the text. We have also clarified in the text that it is the time of NATT start that changes and not time of surgery. This was missing, thanks.

In Figure 3A, the NATT duration for the PT part is the same for all three panels. The purpose of Figure 3A (for PT kinetics) is to show the impact of dose, and dose only. The impact on MB is depicted using the color gradients within each panet. Figure 3B shows the impact of varying NATT duration, for the three dose levels of the three columns. We hope the Figure is clearer now.

Errors, typos and suggestions for phrasing

• The second to last sentence in the abstract is not that clear, it is better phrased at the end of the introduction (L50-52).

Thanks for the suggestion, the abstract has been clarified accordingly.

• In the Major findings section, the term RTKI is not explained. In fact, the abbreviation is not explained anywhere, it rather seems to be used synonymously for “VEGFR TKI”. Please stick to only one of these terms.

Agreed and modified throughout text.

• In the first paragraph of the Introduction, two topics are mixed up, namely neoadjuvant trials and breast cancer. Please introduce them separately first, and only then discuss neoadjuvant trials for breast cancer.

Since our entire study relies on experimental data from (triple negative) breast cancer, and given that neoadjuvant treatment is particularly relevant to breast cancer, we prefer to stick to being specific to breast cancer.

• L 5: “faster to conduct” compared to what?

Compared to adjuvant trials or trials in the metastatic setting, because the primary endpoint of the latters is often overall survival requiring long-term follow-up, whereas neoadjuvant trials have endpoints requiring a shorter time frame (e.g., pathologic complete response).

We have made this sentence more precise.

• L24 remove “which we termed mu”, it is not helpful in the introduction.

Agreed.

• L26 it should be “hypotheses”, not “hypothesis”

Thanks. Corrected.

• L89 “tumor” missing after “primary”

Thanks. Corrected.

• L149 it should be Ki67 instead of Ki76

Indeed. Thanks, corrected.

• L157, in the second argument of the min function, “*v” is missing.

Indeed. Thanks, corrected.

• L208 use “model prediction” instead of “model value”

Done

• The error models in L 210 and L 215 contradict each other. Please rewrite or remove L 210 for consistency.

Correct. Thanks for noticing. We have modified accordingly.

• L405 do you mean “could be considered underwhelming”?

We do mean "could be underwhelming", i.e., that despite positive results in preclinical testing, results in the clinical setting have sometimes been disappointing.

• L408: I was irritated by the phrasing “with no fitting involved” since in L201-203, it is stated that model parameters were estimated using a mixed-effect model.

We apologize if there was a confusion but these results were indeed obtained "*with no fitting involved*", in the sense that none of the data was used to calibrate the model (identify the parameters), during this first phase of investigation. We have clarified the methods and results to better explain. We first used the parameters from a previous calibration to untreated animals (ref. 3), and heuristically modeled the treatment effect by setting either $g_{p}$ (scenario A) or $g_{p}$ and $g$ (scenario B) to zero during NATT. Thus, model simulations in Figure 1 are true predictions.

• There’s an error in the middle panel of Fig 3. Surgery should be at the second, not first dashed line.

Indeed, thanks. This has been corrected.

• Also in Fig 3, color coding of axes and lines would make panel B easier to read.

Thanks for the suggestion but we believe panel B, although not straightforward, is possible to understand.

• Residual variability parameters for primary tumor and metastases are missing in Table 1

Added.

• Caption Tab S1, “Doeses” should be “Doses”

Corrected.

• In Fig S5, please explain the color-coding: left column primary tumor, right column metastases (even though it is used consistently everywhere).

Done.
