# Supplementary material for: Machine-learning and mechanistic modeling of metastatic breast cancer after neoadjuvant treatment
Source: PLoS Comput Biol. 2024 May 3;20(5):e1012088. doi: 10.1371/journal.pcbi.1012088 (PMC11095706; doi:10.1371/journal.pcbi.1012088)
Supplement: Attachment — Submitted filename: rebuttal_2.docx [file pcbi.1012088.s012.docx]

Sebastien Benzekry, PhD

Head of the Inria – Inserm team COMPO

COMPutational pharmacology and clinical Oncology

Centre Inria Sophia Antipolis – Méditerranée

Centre de Recherches en Cancérologie de Marseille

Inserm U1068, CNRS UMR7258, Institut Paoli-Calmettes

Pharmacy faculty, Aix-Marseille University

**Pr P. Maini**

**The editorial office**

**PLoS Computational Biology**

Marseille, February 27, 2024

Dear Editor,

Please find below a response to the remaining comments of Reviewer 2.

We would also like to point that we made public the entire data generated for the study at <https://zenodo.org/records/10607753>. This comprehensive dataset contains pre-surgical primary tumor volume measurements and pre- and post-surgical records of metastatic burden in 251 mice implanted with human breast cancer cells either untreated or pre-surgically treated with two distinct receptor tyrosine kinase inhibitors (Sunitinib and Axitinib) and multiple dose and scheduling regimen.

Sincerely,

Dr Sebastien Benzekry, Corresponding Author.

Reviewer 2

The authors submitted a revised version that answered most of my requests. However, there are still some two issues that need to be addressed.

Comment 4

The authors have moved their definition of metastatic relapse to the Methods section as requested, but unfortunately, they didn’t address the second part of my request. It is still not stated which threshold value (in terms of metastatic burden) results from their rationale. All I can see is that, from Fig S2 (Vehicle), the threshold is somewhere around 10^8 cells. But since only 10th, 50th and 90th percentiles are drawn in Fig S2 (and no 30th percentile, which enters the relapse definition), this can only be guessed. Please add the exact numeric threshold value to the Results.

Comment 6

Unfortunately, the explanations and revisions regarding this point are not at all satisfying. The authors merely changed the word “Tx” to “NATT” in Figure 3 without addressing my concerns.

We thank the reviewer for this comment and apologize for not fully addressing these issues. Our revised manuscript now includes changes in response to each topic.

First, if, as the authors state in the rebuttal, the time of surgery is fixed at 45 days, then the gradient in the metastatic burden as a function of NATT should be inverted, i.e. higher burden for NATT = 0 days than for 18 days.

You a correct. In fact, it is not the time of surgery that is fixed but rather the time of NAT initiation as it is assumed to be the time of detection of the primary tumor. Thus, the gradient is correct but text was not. Our revised manuscript now includes the following modifications to the text:

*" The NAT initiation time was fixed to 27 days, assumed to be the time of detection of the primary tumor. The resection time fixed was then varied from 27 to 45 days. Three dose levels were simulated (60, 120 and 240 mg/kg, see Fig 3)."*

Second, it is very confusing to present a figure in which the primary tumor burden is shown for one NATT duration, while the metastatic burden is shown for different ones, in the same panel. There should be one PT burden curve per NATT duration, decreasing from different time points onwards. Taking these two points together, it seems possible that the simulations were carried out in a different way: fixed start of NATT, but different times of surgery. This would explain both the gradient in metastatic burden and the overlapping primary tumor burden.

On this point the reviewer is correct. In our first version of the manuscript we had included a note about this in the figure legend, which read "*Primary tumor growth curves are not distinguishable because they are all superimposed until time of surgery.*"

However, even in this scenario, there should be some graphical hint in the left part of the panels that NATT durations differ.

We agree and modified the Figure to have a color gradient also present in the PT growth curves. The modified figure is shown below.

Finally, annotations are still not used consistently. In the legend of Fig 3B, a reference is made to the survival model. Also, the terms Tx / NAT / NATT are still mixed up throughout the manuscript/captions/figures.

We thank the reviewer for this comment and agree that our manuscript was not clear in sections or consistent in how terms were defined and presented. To correct, this we have undertaken edits to the abstract and introduction where we more consistently presented the novelty of the study and the terms used. In the manuscript, we have been clearer with our use of the term for neoadjuvant therapy (NAT) and for references to pre-surgical primary tumor (PT) as well as post-surgical metastatic burden (MB). We hope these changes have improved the manuscript and made it for understandable for readers.
