# Supplementary material for: Machine-learning and mechanistic modeling of metastatic breast cancer after neoadjuvant treatment
Source: PLoS Comput Biol. 2024 May 3;20(5):e1012088. doi: 10.1371/journal.pcbi.1012088 (PMC11095706; doi:10.1371/journal.pcbi.1012088)
Supplement: Attachment — Submitted filename: rebuttal_3.docx [file pcbi.1012088.s013.docx]

Sebastien Benzekry, PhD

Head of the Inria – Inserm team COMPO

COMPutational pharmacology and clinical Oncology

Centre Inria Sophia Antipolis – Méditerranée

Centre de Recherches en Cancérologie de Marseille

Inserm U1068, CNRS UMR7258, Institut Paoli-Calmettes

Pharmacy faculty, Aix-Marseille University

**Pr P. Maini**

**The editorial office**

**PLoS Computational Biology**

Marseille, April 7, 2024

Dear Editor,

Reviewer 1 had no further comment. Please find below the response to reviewer 2.

Reviewer 2

The authors have appropriately addressed the two remaining issues that I raised in the last round of revision. However, I still cannot see any computational code submission -- only the experimental data are stored under the Zenodo link.

We have released the code publicly at <https://gitlab.inria.fr/benzekry/metamats.burden.treatment>. To ensure long-term reproducibility of the results, we also provide a software heritage identifier permalink for a code snapshot. This ensures that any future modification to the code won't alter the reproducibility, and that the code behind the paper will remain accessible in the long-term.

We would also like to point to the attention of reviewer 2 that we give all the details in the generation of the Figures. Specifically, the code of Figure 1 shows that plots in Figure 1C were true predictions on new data, using parameters that had been calibrated from a previous, distinct study on untreated animals and reported in (Benzekry et al., Cancer Res, 2016).

Sincerely,

Dr Sebastien Benzekry, Corresponding Author
